# Apple Peel Supplemented Diet Reduces Parameters of Metabolic Syndrome and Atherogenic Progression in ApoE−/− Mice

**DOI:** 10.1155/2015/918384

**Published:** 2015-05-17

**Authors:** Jaime Gonzalez, Wendy Donoso, Nathalie Sandoval, María Reyes, Priscila Gonzalez, Monica Gajardo, Erik Morales, Amalia Neira, Iván Razmilic, José A. Yuri, Rodrigo Moore-Carrasco

**Affiliations:** ^1^Departamento de Bioquímica Clínica e Inmunohematología, Facultad de Ciencias de la Salud, Universidad de Talca, P.O. Box 747, Talca, Chile; ^2^Programa de Magister en Ciencias Biomédicas, Facultad de Ciencias de la Salud, Universidad de Talca, Chile; ^3^Departamento de Estomatología, Facultad de Ciencias de la Salud, Universidad de Talca, Chile; ^4^Instituto de Química y Recursos Naturales, Universidad de Talca, Chile; ^5^Facultad de Medicina, Universidad Católica del Maule, Avenida San Miguel 3605, 3480112 Talca, Chile; ^6^Centro de Pomáceas, Facultad de Ciencias Agrarias, Universidad de Talca, Chile; ^7^Centro de Estudios en Alimentos Procesados (CEAP), Conicyt-Regional, Gore Maule, R09I2001, Avenida San Miguel 3425, 3480137 Talca, Chile; ^8^Programa Investigación de Excelencia Interdisciplinario en Envejecimiento Saludable PIEI-ES, Universidad de Talca, Avenida Lircay s/n, Talca, Chile

## Abstract

Cardiovascular Diseases (CVD) represent about 30% of all causes of death worldwide. The development of CVD is related in many cases with the previous existence of metabolic syndrome (MS). It is known that apple consumption has a cardiovascular protecting effect, containing phenolic compounds with antioxidant effect, which are concentrated in the fruit peel. The objective of this study was to test the effect of apple peel consumption in a murine model of MS and apoE−/− mice. Apple supplemented diets reduced the biochemical parameters (glycaemia, total cholesterol, HDL-cholesterol, LDL-cholesterol, ureic nitrogen, triglycerides, insulin, and asymmetric dimethylarginine (ADMA)) of MS model in CF1 mice significantly. The model apoE−/− mouse was used to evaluate the capacity of the apple peel to revert the progression of the atherogenesis. FD with HAP reverts cholesterol significantly and slows down the progression of the plate diminishing the cholesterol accumulation area. With these results, it can be concluded that the consumption of apple peel reduces several MS parameters and the atherogenic progression in mice.

## 1. Introduction

Cardiovascular diseases (CVD) are the main cause of death in the world [1] and there are several associated risk factors, among them, metabolic syndrome (MS), characterized by hyperglycemia, dyslipidemia, arterial hypertension, and obesity of central distribution.

The rate of atherothrombotic progression is influenced by the exposition to risk factors which increase the probability to suffer from a cardiovascular event. Such factors can be classified as modifiable (smoking, hypertension, diabetes, dyslipidemia, and sedentarism) and nonmodifiable (sex, age, and genetic). For this reason, genetically modified experimental animals have been generated which have contributed to the knowledge of the atherothrombotic phenomenon: the formation of the atheroma plaque, the induction of its formation, and the cellular elements involved [2].

Epidemiologic studies have demonstrated that fruit and vegetable consumption contributes to decreasing the cardiovascular risk [3]. For instance, apple consumption has been inversely associated with acute myocardial infarction [4]. It has been described that the protecting effect of apple consumption has different levels and mechanisms of action: body weight loss and reduction in glycemia [5, 6], protective against low density lipoprotein oxidation (LDL)* in vitro* [7, 8], antiadipogenic, hypocholesterolemic, and antiatherogenic effects [9, 10], and inhibition of cholesteryl ester transfer protein activity, improving the distribution of cholesterol in lipoproteins [11]. Studies* in vitro* have demonstrated that apple flavonoids improve the availability of nitric oxide and protect the endothelial cells from apoptosis preventing the process of endothelial dysfunction and* in vivo* reduces blood pressure and oxidative stress [12]. Furthermore, flavonoids interfere with the proliferation and migration of smooth muscle cells preventing the association of them with others that will initiate the atherothrombotic process [13]. In the apple, polyphenols content and the level of antioxidant activity are about five times higher in peel than in pulp [14].

The ApoE deficient mice are an idoneous experimental model for the study of atherosclerosis [15], because it is unable to carry out the reverse cholesterol transport, since it lacks the gene coding for apolipoprotein E. The lack of this gene involves accumulation of lipids at the luminal level in blood vessels, developing lesions in the microvasculature and showing total blood cholesterol levels ≥500 mg/dL, mostly VLDL and remaining chylomicrons, after administering a normal diet [16]. The atherogenic process can be accelerated or induced in this model through the administration of high fat diets, allowing studying the process in a model different from human, with significant similarities [17]. In this study, we aimed to evaluate different forms of consumed apple skin: fresh skin, which is the primary product, dehydrated skin, representing a useful alternative in the conservation and transport of this product, and sunburned skin, which contains a higher amount of antioxidants, but its potential effect has not been demonstrated in biological assays [18]. For this reason we used CF1 mice fed a high fat diet, a model of early stage of CVD, and ApoE knockout mice, a model of atherosclerosis, to study the impact of apple peel consumption.

## 2. Materials and Methods

### 2.1. Animals

Four-week male CF-1 mice and C57BL6 mice obtained from the Public Health Institute, Santiago, Chile, and ApoE−/− mice (donated by Dr. Atilio Rigotti, Pontificia Universidad Católica de Chile) were used in the experiments. Animals were maintained at 22 ± 2°C, at a regular darkness-light 12:12 h cycle (light 08:00 to 20:00 h). Weight, food intake, and water consumption were measured daily. Animal handling was carried out in conformity with the regulation for the use of laboratory animals of the National Commission for Scientific and Technologic Research (CONICYT), Chile. The protocol was approved by the bioethics committee of Universidad de Talca. After an adaptation period of 10 days, animals were randomized into 5 groups.

### 2.2. Anesthesia and Euthanasia

At the end of the experimental period, mice were anesthetized with an intraperitoneal injection of ketamine 50 mg/kg (Ketostop; DrogasPharma-Invetec, Santiago, Chile) and xylazine 5 mg/kg (Xylavet; Alfasan International BV, Holland). Animals were euthanatized by blood collection from the abdominal aorta and later rupture of the diaphragm. Plasma and tissues were harvested and stored at −70°C until the analysis.

Initial and final weight of each animal, as well as systolic arterial blood pressure (SAP) at the tip of the mice tails, were obtained by using ultrasound equipment (Ultrasonic Doppler Flow 811-B, Aloha, Oregon, USA).

### 2.3. Diets

CF-1 mice were administered five types of diet: normal diet (ND), fat diet (FD), fat diet plus healthy apple peel (FD + HAP), fat diet plus dehydrated healthy apple peel (FD + DAP), and fat diet plus sun damaged apple peel (FD + SDAP) coming from Fuji apples, during 43 days.

ApoE−/− mice were administered ND, FD, and FD + HAP during 10 weeks using peel from Granny Smith apples as a supplement. C57BL6 mice were administered ND. Ingredients used and the preparation of different diets were performed as described by Moore-Carrasco et al., 2008 [19]. Different types of apple peel were added to ND and FD in 20% w/w. For the analysis of diet components, three pellets from each of them were selected at random and submitted to the Institute of Chemistry of Universidad de Talca for chemical analysis according to official methods of analysis of the Association of Official Analytical Chemists [20]. The lipid content of fat fraction was determined by mass spectrometry/gas chromatography (Perkin Elmer Turbo Mass and Autosystem XL Gas Chromatograph). Antioxidant capacity was determined measuring free radical traps 2,2-diphenyl-1-picrylhydrazyl by the method described by Von Gadow et al. (1997) [21] with modifications, using chlorogenic acid as standard. Glycoside quercetins were determined by High Performance Liquid Chromatography/Diode Array Detector (Merck Hitachi, LaChrom, Tokyo, Japan).

#### 2.3.1. Determination of Total Phenolics Concentration

Total phenolics were determined by the Folin-Ciocalteu method [22]. Briefly, 0.1 mL of extract was mixed with 0.5 mL of the Folin-Ciocalteu phenol reagent (Merck, Darmstadt, Germany). The mixture was incubated for 5 min and then 0.5 mL of sodium carbonate (Na2CO3; 10%, w/v) was added and incubated for 15 min at room temperature. Absorbance was measured at 640 nm in a spectrophotometer. Total phenolic concentrations in the peel and diets were expressed as mg of chlorogenic acid equivalents (CAE) g-1 FW.

#### 2.3.2. Determination of Antioxidant Activity

The capture of the free radical 2,2-diphenyl-1-picrylhydrazyl (DPPH; Fluka Chemie, Buchs, Switzerland) was measured by the method described by Von Gadow et al., [21] with modifications. Briefly, 0.1 mL extracts were mixed with 2 mL of 8 × 10^−5^ M DPPH solution and incubated for eight min at room temperature and the absorbance measured at 515 nm. Ethanol was used as blank. Chlorogenic acid in different concentrations was used as a standard and the capture of the DPPH free radicals was expressed as mg of chlorogenic acid equivalents (CAE) g-1 FW.

#### 2.3.3. Determination of Specific Phenolics by HPLC

Specific phenolics (quercetins glycosides) and lipid profile in the samples (diets) were determined using a HPLC-DAD Merck Hitachi (LaChrom, Tokyo, Japan) equipment consisting of a LaChrom L-7100 pump and a diode array detector, L-7455 LaChrom, and a 100-5 C18 Kromasil column of 259 mm × 4.6 mm with a precolumn of the same characteristics, maintained at 20°C. Briefly, 0.02 mL previously filtered (0.45 *μ*m filter) extracts were injected. To identify the compounds, different standards of specific phenolics were used with the UV-VIS spectra. The chromatogram was monitored at 256 nm. The solvents of the mobile phase were A: 1% formic acid in H_2_O quality HPLC, B: 40% acetonitrile in H_2_O, and C: acetonitrile. The elution parameters were time 0–10 min: A (70), B (30), and C (0) flow 1 mL min^−1^; time 45 min: A (25), B (75), and C (0) flow 0.5 mL min^−1^; time 52 min: A (0), B (0), and C (100) flow 1 mL min^−1^; and time 55 min: A (70), B (30), and C (0) flow 1 mL min^−1^. The results were expressed in *μ*g of samples in g of FW-1.

### 2.4. Biochemical Parameters

Total cholesterol, HDL cholesterol, triglycerides, transaminase GOT, and glucose were determined by enzymatic and standardized colorimetric methods.

### 2.5. ELISAs

Plasmatic concentrations of insulin (ELISA Kit Rat/Mouse Insulin, Millipore) and asymmetric dimethylarginine, ADMA (ADMA Direct (mouse/rat) ELISA Kit, Immundiagnostik), were determined by ELISA. Measurements were obtained following the instructions of each manufacturer.

### 2.6. Histology

Thoracic aorta obtained from ApoE−/− and C57BL6 (Wild Type, WT) mice were fixed in 10% formalin at pH 7.4. Once the samples were fixed, the dehydration, clarification, and inclusion protocol were carried out. After blocks were obtained, sections were obtained using a microtome (Microm HM325), with a thickness of 5 *μ*m. Sections of hydrated and deparaffinized tissues were stained with hematoxylin and eosin (HE) and Masson's Trichrome, following the appropriate methods for histologic observation. From each aorta description, 10 sections were analyzed by 3 independent observers (J.M., E.M., and R.MC.).

### 2.7. Image Processing

After the staining procedures, photographs (40x) were obtained with a Primo Star Carl Zeiss trinocular microscope used for this purpose and a Canon EOS Rebel XSI camera with the software EOS Utility (version 2.4, Copyright^©^ CANON INC, USA, 2008) was used for image capturing. Later, they were quantified to determine the indirect presence of lipids as well as the degree of inflammation of the lesions, in terms of the presence of inflammatory cells; determining representative areas in each artery lesion with a dimension of 50 *μ*m^2^, the software AxioVision Release 4.8.1 (Carl Zeiss version 11. 2008) was used for this purpose, and the software Micrometrics SE Premium (version 2,8; ACCU-SCOPE Inc., Commack, NY, USA, 2008) was used for counting inflammatory cells. The lesion surface was quantified by using the ImageJ software (version 1,46j; National Institute of Health, USA, 2006) to determine the degree of fibrosis. Each image was analyzed through the Colour Deconvolution Plugin, selecting the vector for Masson's Trichrome, determining the quotient (density/area), and allowing the characterization of each lesion [23]. From each of the image analyses, 10 sections were measured by 2 independent operators (J.M. and W.D.).

### 2.8. Statistical Analysis

The results are presented as means ± standard deviation (SD). Comparisons between groups were done by the analysis of variance (ANOVA) using Graphpad software. A statistical difference was evaluated using a confidence interval of 95%, with Dunnett's and Newman-Keuls as post hoc tests.

## 3. Results

### 3.1. Nutritional Composition of Diets

First, we prepared diets supplemented with apple products.  Table 1 shows the analysis of each diet administered to mice, in which, according to the results, ND can be homologated to a human diet since they have similar percentage of several components, while the rest of the diets have higher content of fat. These, except FD + HAP, present low percentage of myristic acid. While all diets have high content of oleic, linoleic, and palmitic acids and low contents of stearic, palmitoleic, and myristic acids, ND and FD do not have palmitoleic acid methyl ester (Table 1).  Table 1 shows the content of total phenols and antioxidant activity of different diets. A low (nondetectable) content of phenols in ND and FD is observed compared with higher content of phenols and antioxidant activity in FD + HAP, FD + DAP, and FD + SDAP.  Table 1 shows the quercetin content (Q); in general, a higher content of different Q in FD + HAP, FD + DAP, and FD + SDAP is observed. Diet supplemented with SDAP shows the highest content of all evaluated quercetins, except Q. rutinoside (Table 1).

### 3.2. Impact of Apple Consumptions in CF-1 Mice: A MS Model

Next, we tested our diets in an animal model of MS, for 43 days.  Table 2 shows the body weight of mice, at the beginning and at the end of the experimental period. Mice subjected to FD present a higher weight gain (7.49 ± 0.35 g) than the ND group (6.90 ± 0.12 g, *P* < 0.01). On the other hand, mice subjected to FD + HAP, FD + DAP, and FD + SDAP presented a lower weight gain when compared to the FD group.

**Figure 1:**
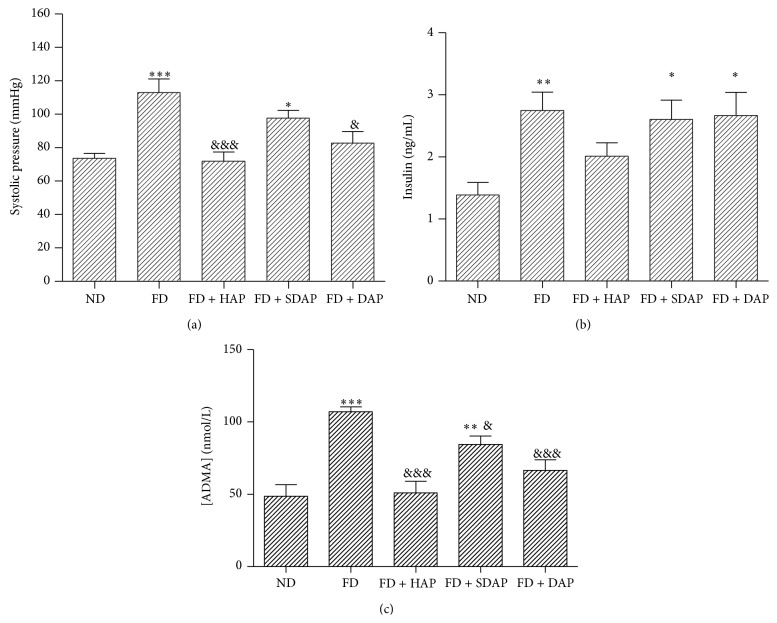
(a) Systolic blood pressure of mice subjected to different diets. ND, normal diet; FD, fat diet; HAP, healthy apple peel; SDAP, sun damaged apple peel; DAP, dehydrated apple peel. Statistical significance: ^∗^ *P* < 0.05, ^∗∗∗^ *P* < 0.001 versus normal diet. Statistical significance: ^&^ *P* < 0.05, ^&&&^ *P* < 0.001 versus fat diet. All groups have a minimum of 4 mice. (b) Average insulinemia in mice fed with different diets. Statistical significance: ^∗^ *P* < 0.05, ^∗∗^ *P* < 0.01 versus normal diet. Statistical significance: ^&^ *P* < 0.05 versus fat diet. All groups have a minimum of 4 individuals. (c) Serum ADMA of mice fed with different diets. ^∗∗^ *P* < 0.01, ^∗∗∗^ *P* < 0.001 versus normal diet. Statistical significance: ^&^ *P* < 0.05 versus fat diet. All groups have a minimum of 4 individuals.

Regarding blood pressure, the group of mice that received FD presented significantly higher SAP (111 ± 1.4 mmHg, *P* < 0.01) than mice subjected to ND (79.14 ± 10.1 mmHg) (Figure 1(a)). On the other hand, mice subjected to FD + HAP, FD + DAP, and FD + SDAP presented a significantly lower SAP than mice subjected to FD. The FD + HAP group was the one which presented lower SAP (*P* < 0.01).

Mice that received FD presented high levels of blood cholesterol (164 ± 17 mg/dL, *P* < 0.05 versus ND), triglycerides (171 ± 3 mg/dL, *P* < 0.01), and glycemia (343 ± 2.0 mg/dL, *P* < 0.01) when compared to mice subjected to ND (102 ± 3.0; 106 ± 6.0; 311 ± 5.5 mg/dL, resp.) (Table 2). Mice subjected to FD + HAP, FD + DAP, and FD + SDAP presented lower levels in these parameters when compared to the FD group. The FD + HAP group (134 ± 30 mg/dL, *P* < 0.001) was the one that presented lower levels of glycemia and the group FD + SDAP apple was the one which presented lower levels of triglyceridemia (74 ± 8.3 mg/dL, *P* < 0.001), when compared to FD group.

The group of mice that received FD (2.34 ± 0.37 ng/mL) presented significantly higher insulin levels than mice fed with ND (1.23 ± 0.25 ng/mL) (Figure 1(b)). On the other hand, mice subjected to FD + SDAP presented significantly lower insulinemia values (1.53 ± 0.32 ng/mL) when compared to FD group.

The group of mice subjected to FD presented higher serum ADMA levels than mice subjected to ND (*P* < 0.001) (Figure 1(c)), while mice fed with FD + HAP, FD + DAP, and FD + SDAP presented lower concentrations than FD group (*P* < 0.001; *P* < 0.01; *P* < 0.01, resp.), the FD + HAP group being the one that presented the lower ADMA levels.

### 3.3. Impact of Apple Consumptions in ApoE−/− Mice: A Model of Atherosclerosis

Next, we tested our diet with apple peel supplement in the ApoE−/− mouse, a model of dyslipidemia and atherosclerosis. Total cholesterol, triglycerides, and basal glycemia were determined in mice fed with the three types of diets, and control group of C57BL6 (WT) mice were incorporated for this analysis. Total cholesterol of ApoE−/− mice fed on a ND (*P* < 0.01), FD (*P* < 0.001), and FD + HAP (*P* < 0.001) was higher than control group (WT). Only glycemia of FD group had a significant increase with respect to the control group (*P* < 0.05).

Total cholesterol of the FD group and the FD + HAP group had statistically significant increase with respect to the ND group (*P* < 0.01), and only basal glycemia of the FD group had a statistically significant difference compared to the ND group (*P* < 0.05) (Table 3).

The final weight was higher in FD group than in FD + HAP group, with a value of *P* < 0.05 (Table 3).

Besides, using ANOVA to compare the initial and final body weight of all groups, it could be determined that the increase in body weight of mice on FD and mice on FD + HAP compared to the initial body weight of mice on ND was statistically significant (*P* < 0.0001). Also, mice on FD had higher body weight than mice on FD + HAP (*P* < 0.05).

### 3.4. Atherosclerosis

**Figure 2:**
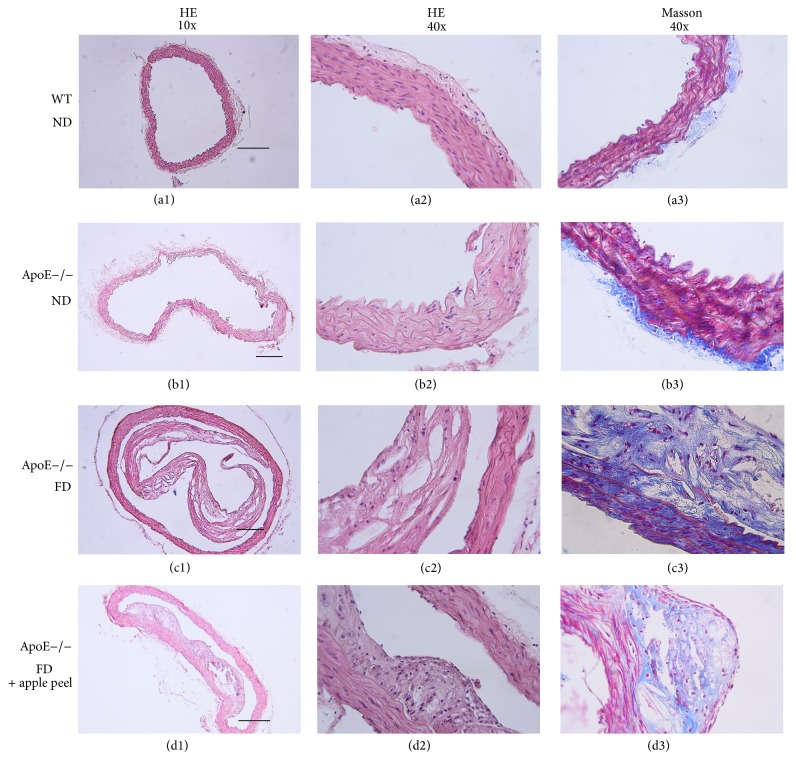
Aorta histochemical staining in mice: (a1) to (a3) control group C57BL6WT; (a1): Hematoxylin and eosin (HE) staining, magnification 10x; (a2): HE staining, magnification 40x; (a3): Masson's Trichrome staining, magnification 40x. (b1) to (b3): ApoE−/− normal diet group: (b1): HE staining, magnification 10x; (b2): HE staining, magnification 40x; (b3): Masson's Trichrome staining, magnification 40x. (c1) to (c3): ApoE−/− fat diet group: (c1): HE staining, magnification 10x; (c2): HE staining, magnification 40x; (c3): Masson's Trichrome staining, magnification 40x. (d1) to (d3): ApoE−/− fat diet plus apple peel group: (d1): HE staining, magnification 10x; (d2): HE staining, magnification 40x; (d3): Masson's Trichrome staining, magnification 40x. Scale bar: 200 *μ*m.

Finally, we analysed the progress of the atherosclerosis process in ApoE−/− mice fed with apple peel supplemented diets for 20 weeks. For this, we collected the thoracic aorta from animals treated and untreated and they were processed for HE and collagen staining.  Figure 2 depicts representative histologic sections of HE and Masson's Trichrome from ApoE−/− mice.

Figures 2(a1) and 2(a2), as well as Figures 2(b1) and 2(b2), correspond to control groups (wild type (WT) and ApoE−/− fed with ND) showing histological sections corresponding to thoracic aorta, in which a vascular wall of conserved histoarchitecture is observed, with inner, middle, and adventitia layers without morphological evidence of lesion.

Figures 2(a3) and 2(b3) show histological sections of control groups WT and ApoE−/− with ND, corresponding to thoracic aorta, stained with Masson's Trichrome technique. A vascular wall of conserved histoarchitecture is observed, with inner, middle, and adventitia layers without morphological evidence of lesion.

Figures 2(c1), 2(c2), and 2(c3) are histological sections of aorta artery of ApoE−/− mice fed with FD corresponding to an elastic artery wall. Figures 2(c1) and 2(c2) were stained with HE technique and Figure 2(c3) was stained with Masson's Trichrome technique. A vascular wall presenting a high degree of histoarchitecture distortion, significant expansion, and partial detachment of the inner layer is observed.  Figure 2(c2) highlights the composition of the histoarchitecture distortion, with abundant foam cells, some inflammatory cells, and abundant optically empty spaces compatible with cholesterol crystals.  Figure 2(c3) highlights degenerative changes of the extracellular matrix at the level of the distortion area.

**Figure 3:**
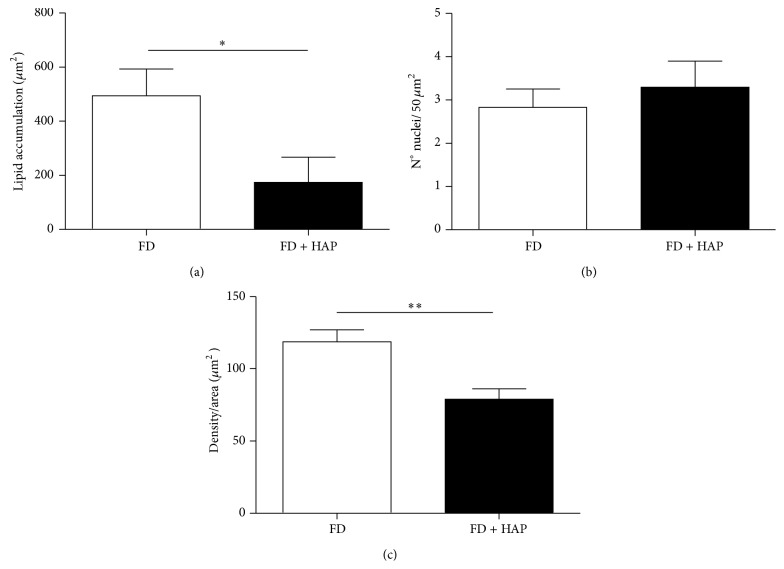
Image analysis of atherosclerotic lesions in ApoE−/− mice. (a) shows the area of cholesterol expressed as *μ*m^2^ of the lesion. In (b), the degree of infiltration expressed as number of cores in the lesion is shown. (c) illustrates the degree of infiltration expressed by the color intensity of the area. Statistical significance: ^∗^ *P* < 0.05, ^∗∗^ *P* < 0.01 versus fat diet.

The image analysis is presented in Figure 3 for the FD and FD + HAP groups. With the images obtained by HE staining, areas of 50 *μ*m^2^ were delimited and the empty area compatible with cholesterol accumulation was measured. The results show that animals fed with a fat diet supplemented with apple peel developed lower size plaques with lower cholesterol content than controls which consume only FD. In the same groups of images and sections, the amount of inflammatory cell nuclei and the intensity of blue colouring in Masson's Trichrome staining were measured. These results show that animals receiving apple peel supplement developed lower degree of fibrosis than animals on fat diet.

## 4. Discussion

### 4.1. MS Mice Model

Murine models for MS research are widely used. Moore-Carrasco et al. standardized a MS model in CF-1 mice fed with a high fat diet and developed metabolic alterations similar to those observed in human MS [19].

MS corresponds to a series of metabolic alterations that double the risk of suffering CVD [24]. The alteration of parameters, such as glycemia, insulin, triglycerides, and arterial pressure, in addition to the increase of prothrombotic molecules such as TNF*α*, ADMA, and P-selectin, contributes to the development of this pathology. The consumption of flavonoids as those present in apples might contribute to the decrease in cardiovascular risk factors. To evaluate this hypothesis, the development of MS was induced in CF-1 mice with a fat-rich diet for a period of 43 days [19] and the effect of different types of Fuji apple peel was determined. Here we showed that the consumption of a hyper caloric diet supplemented with fresh, dehydrated, or sunburned apple peel prevents the metabolic and hormonal alterations triggered by the MS. For instance, apple peel supplemented diets reduced blood glucose levels while they also reduced insulin levels in CF-1 mice. These results suggest a role in glucose metabolism. The mechanism for this improvement remains to be determined but may involve improved insulin sensitivity, decreased intestinal glucose absorption, or decreased hepatic glucose output.

The composition of lipids present in the different types of diets used for this study is very similar to other diets used in previous studies [25]. The murine model developed by our group [19] was validated in this study; when subjecting CF-1 mice to a diet rich in fat (17%) for a 43-day period, they developed MS.

Mice fed with FD presented a significantly higher weight gain when compared to mice subjected to ND. Mice fed with FD + HAP, FD + DAP, and FD + SDAP presented a lower weight gain than the group fed only with FD. These results agree with a study by Conceição de Oliveira et al. (2003) [26].

Mice that received FD show higher SAP than mice fed with ND. Mice subjected to FD+ HAP showed lower SAP compared to FD. These results are in agreement with other* in vivo* studies that have demonstrated that flavonoids present in apple decrease blood pressure [12] and with a study in a model of hypertensive rats in which antioxidants, such as the quercetins present in apples, decrease arterial pressure [27]. Recently, the capacity of a beverage prepared from fruits (cranberry, apple, and blueberry juices at the portions of 12.5%, 37.5%, and 50%, resp.) was described to reduce blood pressure in rats [28].

Mice that consumed a FD showed an increase in glycemia, triglycerides, and cholesterol when compared to the ND group, while mice subjected to FD + HAP, FD + DAP, and FD + SDAP showed significantly lower levels of these parameters compared to the FD group. These results agree with the study by Zhao et al. (2004) [29], using transgenic mice for type II diabetes. They demonstrated that the treatment with phloridzin, a component which is present in apple peel, reduced significantly the hyperglycemia in these mice. Aprikian et al. [30] evidenced a significant decrease of cholesterol levels in rats fed with a high fat diet complemented with lyophilized apple; the same was observed in obese Zucker rats [30]: the consumption of apples decreased the plasmatic LDL cholesterol [31].

The group of mice that received FD showed insulin levels significantly higher than the control group, and mice with FD + HAP showed insulin values significantly lower compared to the FD group. Gao et al. (2010) [32] demonstrated that mice fed with a high fat diet developed obesity and hyperinsulinemia, in only two weeks.

Studies have shown that the intake of polyphenol-rich foods influences cardiovascular health and decreases the expression of P-selectin [33]. Also, it has been observed that quercetins reduce significantly the concentrations of this adhesion molecule [34]. However, in this study, we did not find significant differences of P-selectin levels among groups (data not shown). It is known that platelet activation followed by inflammatory stimuli leads to the expression of surface receptors as P-selectin. Endothelial dysfunction, an early characteristic in atherosclerosis, is associated with the low degree inflammation inside the vascular wall [35]. This might explain partly the results obtained in this study in regard to P-selectin, insomuch as mice developed MS which causes a low degree of inflammation appearing usually in early stages of atherosclerosis.

We found that mice subjected to FD showed higher ADMA levels when compared to the ND group, which indicates that endothelial dysfunction is associated with MS. Mice subjected to FD + HAP, FD + DAP, and FD + SDAP showed lower plasma concentrations of ADMA compared with the FD group, specially the FD + HAP group. Some authors have stated that there is a reciprocal relation between ADMA and insulin resistance, suggesting that when there are metabolic alterations leading to insulin resistance, plasma levels of ADMA increase and vice versa [36, 37]. In this study, mice with FD showed high plasmatic levels of both ADMA and insulin. A study of a group of young adults evidenced a positive association between ADMA plasma levels, obesity, arterial pressure, and glycemia and it was observed that those individuals that consume diets with high content of antioxidants showed significantly lower levels of ADMA [38].

With the data obtained in this study, it can be concluded that an apple peel enriched diet decreased significantly several MS criteria in CF-1 mice.

### 4.2. Atherosclerosis Model

It is well established that raw or processed fruit and vegetable consumption decreases the incidence of CVD [39]. The mechanisms by which apple consumption decreases the CV risk is unknown; however, there is evidence that relates its favourable effect to a high antioxidant content, mostly flavonoids, with peel the main presenting antioxidant activity [14], in which its protecting effect would be related to its capacity to decrease serum cholesterol [40].

In the advanced atherosclerosis model obtained in ApoE−/− mice fed with FD + HAP, we observed a significant decrease of the fat content at the atheroma plaque, expressed as a lipid infiltration area. With these results, we can postulate that the apple peel supplement in diet may have a protecting effect in the advance of the atherosclerotic disease, directly at the fat content level of the plaque, reducing endothelial dysfunction and serum cholesterol. This effect is possibly due to stimulation of cholesterol catabolism, or inhibition of its intestinal absorption, correlating with previous reports in ApoE−/− mice [41], and Sprague-Dawley rats [42]. Flavonoids, mainly from wine, also present in apples have been related to a decrease in migration and proliferation of smooth muscle cells as a response to a decrease of LDL [43]. In our study we observe a significant decrease of fibrosis in ApoE−/− FD + HAP mice, compared to the control group, confirming the results observed with wine flavonoids. However, this is the first time that this characteristic is proved with apple peel. Finally, the data of inflammation level expressed by the number of inflammatory cells infiltrating the plaque were not statistically significant compared with the control group, maybe because the beneficial effects of flavonoids present in apple peel are mainly related to stopping the progression of plaque formation, by reducing cholesterol accumulation, compared with its effect of improving endothelial dysfunction, corroborating the results mentioned above.

As a conclusion, apple peel consumption improves metabolic alterations associated with a fat-rich diet and also slowed the atherogenesis development, one of the most lethal consequences of a hypercaloric diet. Our results contribute to the concept of having a functional food from apple products with beneficial effects on risk factors for cardiovascular disease [44].

## Figures and Tables

**Table 1:** Nutritional report in percentage of lipid composition, total phenols, antioxidant content, and quercetin content of the different diets employed.

Component	ND	FD	FD + HAP	FD + SDAP	FD + DAP
Carbohydrates (%)	69.4	57.0	59.8	57.6	56.2
Proteins (%)	21.0	17.6	12.5	18.2	17.3
Lipids (%)	1.8	16.8	20.2	16.4	17.5
Water (%)	6.0	15.3	19.4	18.3	12.2
Ash (%)	6.0	5.1	4.8	4.8	5.1
Fiber (%)	2.8	3.5	2.8	3.0	3.9
Myristic acid (14:0)	1.6	0.1	1.1	0.0	0.1
Palmitoleic acid (16:1)	0.0	0.0	1.5	4.6	1.4
Palmitic acid (16:0)	40.0	14.0	14.1	38.9	29.7
Linoleic acid (18:2)	11.6	34.0	31.5	15.5	28.2
Oleic acid (18:1)	29.8	44.4	39.4	32.0	19.0
Stearic acid (18:0)	9.7	7.5	6.5	9.0	7.3
Total phenols	0.0	0.0	1.6 ± 0.5	2.0 ± 0.1	1.8 ± 0.1
Antioxidant activity	0.0	0.0	0.2 ± 0.02	0.3 ± 0.1	0.2 ± 0.02
Q. rutinoside	0.0	0.0	16.4 ± 7.2	19.2 ± 4.3	29.0 ± 1.4
Q. galactoside	0.0	0.0	55.9 ± 9.1	93.8 ± 16.8	46.0 ± 4.5
Q. glucoside	0.0	0.0	10.6 ± 5.8	29.9 ± 5.0	10.2 ± 0.4
Q. xyloside	0.0	0.0	7.9 ± 4.4	16.3 ± 2.8	8.9 ± 0.2
Q. arabinoside	0.0	0.0	15.6 ± 4.6	29.7 ± 5.7	20.9 ± 1.1
Q. rhamnoside	0.0	0.0	10.2 ± 3.4	14.5 ± 3.3	14.1 ± 0.9

ND, normal diet; FD, fat diet; HAP, healthy apple peel; SDAP, sun damaged apple peel; DAP, dehydrated apple peel. All lipid components have a methyl ester conjugation. Total phenols and antioxidant capacity expressed with mg eq. chlorogenic acid/sample g. The quercetin glycoside expressed with *µ*g/sample g.

**Table 2:** Biochemical parameters and initial and final weight and weight gain of the different groups of CF-1 mice subjected to different diets.

Biochemical component	ND	FD	FD + HAP	FD + SDAP	FD + DAP
Total cholesterol (mg/dL)	102 ± 5	164 ± 30^∗^	101 ± 9^&^	103 ± 3^&^	112 ± 7^&^
HDL cholesterol (mg/dL)	56 ± 7	66 ± 13	53 ± 6	50 ± 4	61 ± 9
Total/HDL cholesterol (mg/dL)	1.8 ± 0.1	1.5 ± 0.1	2 ± 0.1	1.8 ± 0.3	1.9 ± 0.1
Triglyceridemia (mg/dL)	106 ± 8	171 ± 5^∗∗∗^	81 ± 9^∗&&&^	74 ± 8^∗&&&^	80 ± 7^∗&&&^
Glycemia (mg/dL)	311 ± 10	343 ± 4	134 ± 30^∗∗∗&&&^	278 ± 28	294 ± 28
GOT (UI/L)	166 ± 48	144 ± 21	94 ± 19	116 ± 16	94 ± 33
Uric acid (mg/dL)	1.8 ± 0.2	2.7 ± 1.0	0.8 ± 0.6^&^	1.2 ± 0.4	1.2 ± 0.6
Initial weight (g)	37.3 ± 1.3	38.0 ± 0.9	37.0 ± 1.4	36.4 ± 1.3	36.5 ± 1.1
Final weight (g)	44.3 ± 0.9	45.1 ± 1.1	45.2 ± 1.3	43.3 ± 1.5	43.2 ± 1.5
Gain (g)	6.9 ± 0.12	7.5 ± 0.35^∗∗^	6.2 ± 0.55^∗&&&^	6.0 ± 0.58^∗&&&^	6.7 ± 0.60^∗^

ND, normal diet; FD, fat diet; HAP, healthy apple peel; SDAP, sun damaged apple peel; DAP, dehydrated apple peel.

HDL: high density lipoprotein and GOT: glutamic-oxaloacetic transaminase.

The results are expressed as the ±SD average. Statistical significance: ^∗^ *P* < 0.05, ^∗∗^ *P* < 0.01, and ^∗∗∗^ *P* < 0.001 are compared with the ND group and ^&^ *P* < 0.05 and ^&&&^ *P* < 0.001 are compared with the FD group.

**Table 3:** Biochemical parameters and initial and final weight in different groups of ApoE−/− mice subjected to different diets.

Biochemical component	WT	ApoE−/− ND	ApoE−/− FD	ApoE−/− FD + HAP
Total cholesterol (mg/dL)	86 ± 9	317 ± 67	533 ± 154^∗∗^	515 ± 132^∗∗^
Triglyceridemia (mg/dL)	55 ± 4	141 ± 16	185 ± 42	146 ± 55
Glycemia (mg/dL)	157 ± 44	161 ± 58	324 ± 128^∗^	231 ± 107
Initial weight (g)	29.1 ± 1.8	32.5 ± 1.9	34.0 ± 2.2	34.1 ± 1.6
Final weight (g)	32.1 ± 1.8	35.1 ± 1.2	43.4 ± 4.3^∗∗∗^	40.1 ± 1.9^∗∗,&^

ND, normal diet; FD, fat diet; HAP, healthy apple peel.

The results are expressed as the ±SD average.

Statistical significance: ^∗∗^ *P* < 0.01, ^∗∗∗^ *P* < 0.001 final weight compared with the ND group and ^&^ *P* < 0.05 compared with the FD group.

Statistical significance ^∗^ *P* < 0.05.
